# Supplementary material for: Development of a Nomogram to Predict the Outcome for Patients with Soft Tissue Sarcoma
Source: Vet Sci. 2023 Mar 29;10(4):266. doi: 10.3390/vetsci10040266 (PMC10146366; doi:10.3390/vetsci10040266)
Supplement: Supplementary file 1 [file vetsci-10-00266-s001.zip › Supplementary Materiak.pdf]

Supplementary Table S1: Example of the R code used to develop the clinical nomogram. This utilised the 'rms' and 'survival' packages in R.

```
#RMS load data and nomogram construction
nomo <- read.csv("Clinical_train.csv", TRUE)
library(rms)
mod.Cox <- cph(Surv(DFI, CENSR) ~Palpnew + Mitoticrate + Necrosis, nomo,surv=TRUE)
ddist <- datadist(nomo)
options(datadist='ddist')
surv.Cox <- Survival(mod.Cox)
nom.Cox <- nomogram(mod.Cox,fun=list(function(x)surv.Cox(1095,x)),funlabel=c("3-year
DFS"),lp=FALSE)
plot(nom.Cox)

#RMS validation of Cox model using validation dataset
nomo_valid <- read.csv("Clinical_valid.csv", TRUE)
fit_valid <- cph(Surv(DFI,CENSR) ~Palpnew + Mitoticrate + Necrosis, nomo_valid,x=TRUE,
y=TRUE)
validate(fit_valid, method="boot", B=40, bw=FALSE, rule="aic",type="residual", sls=.05, aics=0,
force=NULL, estimates=TRUE, pr=FALSE, dxy=TRUE, u, tol=1e-9)

#RMS nomogram of IHC training data
nomo <- read.csv("IHC_test.csv", TRUE)
library(rms)
mod.Cox <- cph(Surv(DFI, CENSR) ~VEGF + Decorin + Mitoticrate + Age, nomo,surv=TRUE)
ddist <- datadist(nomo)
options(datadist='ddist')
surv.Cox <- Survival(mod.Cox)
nom.Cox <- nomogram(mod.Cox,fun=list(function(x)surv.Cox(1095,x)),funlabel=c("3-year
DFS"),lp=FALSE)
plot(nom.Cox)

#RMS validation of Cox model using IHC_validation dataset
nomo_valid <- read.csv("IHC_test.csv", TRUE)
fit_valid <- cph(Surv(DFI, CENSR) ~VEGF + Decorin + Mitoticrate + Age, nomo_valid,x=TRUE,
y=TRUE)
validate(fit_valid, method="boot", B=40, bw=FALSE, rule="aic", type="residual", sls=.05, aics=0,
force=NULL, estimates=TRUE, pr=FALSE, dxy=TRUE, u, tol=1e-9)
```

Supplementary Table S2. Results from statistical analysis of all patient and tumour characteristics used in the Clinical_train dataset.

	Mean	Median	Signif.	HR (95% CI)
**Disease free interval (days)**	764.57	655		
**Recurrence**<br>**Recur = 27 (27%)**<br>**No recur = 75 (73%)**				
**Differentiation**<br>**1 = 54 (53%)**<br>**2 = 38 (37%)**<br>**3 = 10 (10%)**			p= 0.7	1.2 (0.7 - 2.2)
**Mitotic rate**<br>**1 = 80 (78%)**<br>**2 = 14 (14%)**<br>**3 = 8 (8%)**			P = 0.03	1.9 (1.1 - 3.2)
**Necrosis**<br>**0 = 74 (73%)**<br>**1 = 23 (23%)**<br>**2 = 5 (5%)**			P = <0.001	2.8 (1.6 – 5.0)
**Grade**<br>**1 = 70 (69%)**<br>**2 = 24 (24%)**<br>**3 = 8 (8%)**			P = <0.001	2.6 (1.6 - 4.3)
**Age**<br>**3 – 16 years**	9.657	9.5		1.0 (0.9 - 1.2)
**Size**<br>**< 1cm = 6 (6%)**<br>**1-5cm = 57 (56%)**<br>**5cm = 39 (38%)**			P = 0.1	1.0 (0.1 - 5)
**Palpable**<br>**Mobile = 49 (48%)**<br>**Fixed = 53 (52%)**			P = 0.001	4.4 (1.8 - 11.05)

Supplementary Table S3. Results from statistical analysis of all patient and tumour characteristics used in the IHC dataset.

	Mean	Median	Range	Signif.	HR (95% CI)
**Disease free interval (days)**	772	655			
**Recurrence**<br>**Recur = 26 (32%)**<br>**No recur = 56 (68%)**					
**VEGF score**<br>**Low = 43 (52%)**<br>**High = 39 (47%)**				p= <0.001	8.4 (2.9 - 24.4)
**Decorin score**<br>**1 = 26 (32%)**<br>**2 = 22 (27%)**<br>**3 = 34 (42%)**				p= 0.7	
**Differentiation**<br>**1 = 31 (38%)**<br>**2 = 40 (49%)**<br>**3 = 11 (13%)**				p= 0.7	
**Mitotic rate**<br>**1 = 61 (74%)**<br>**2 = 12 (15%)**<br>**3 = 9 (11%)**				p= 0.6	
**Necrosis**<br>**0 = 54 (66%)**<br>**1 = 23 (28%)**<br>**2 = 5 (6%)**				p= 0.003	-<br>1.1 (0.4 - 2.7)<br>7.2 (1.9 - 27)
**Grade**<br>**1 = 46 (56%)**<br>**2 = 27 (33%)**<br>**3 = 9 (11%)**				p= 0.5	
**Age**	9.77	10	12		
**Size**<br>**< 1cm = 2 (2%)**<br>**1-5cm = 45 (55%)**<br>**>5cm = 21 (26%)**				p= 0.2	
**Palpable**<br>**Mobile = 32 (39%)**<br>**Fixed = 45 (55%)**				p= 0.03	-<br>2.7 (1.1 - 8)

Supplementary File S1. Dataset used in the creation of the Clinical Nomogram (Clinical_train.csv)

Supplementary File S2. Dataset used to validate the Clinical Nomogram (Clinical_valid.csv)

Supplementary File S3. Dataset used in the creation of the IHC Nomogram (IHC_test all.csv)
